# Lightweight air-to-air unmanned aerial vehicle target detection model

**DOI:** 10.1038/s41598-024-53181-2

**Published:** 2024-01-31

**Authors:** Qing Cheng, Yazhe Wang, Wenjian He, Yu Bai

**Affiliations:** School of Air Traffic Management, Civil Aviation Flight University of China, Guanghan, 618300 China

**Keywords:** Imaging and sensing, Aerospace engineering

## Abstract

The rapid expansion of the drone industry has resulted in a substantial increase in the number of low-altitude drones, giving rise to concerns regarding collision avoidance and countermeasure strategies among these unmanned aerial vehicles. These challenges underscore the urgent need for air-to-air drone target detection. An effective target detection model must exhibit high accuracy, real-time capabilities, and a lightweight network architecture to achieve a balance between precision and speed when deployed on embedded devices. In response to these requirements, we initially curated a dataset comprising over 10,000 images of low-altitude operating drones. This dataset encompasses diverse and intricate backgrounds, significantly enhancing the model’s training capacity. Subsequently, a series of enhancements were applied to the YOLOv5 algorithm to realize lightweight object detection. A novel feature extraction network, CF2-MC, streamlined the feature extraction process, while an innovative module, MG, in the feature fusion section aimed to improve detection accuracy and reduce model complexity. Concurrently, the original CIoU loss function was replaced with the EIoU loss function to further augment the model’s accuracy. Experimental results demonstrate an enhancement in the accuracy of drone target detection, achieving mAP values of 95.4% on the UAVfly dataset and 82.2% on the Det-Fly dataset. Finally, real-world testing conducted on the Jetson TX2 revealed that the YOLOv5s-ngn model achieved an average inference speed of 14.5 milliseconds per image. The code utilized in this paper can be accessed via https://github.com/lucien22588/yolov5-ngn.git.

## Introduction

Unmanned Aerial Vehicles (UAVs), commonly known as drones, are autonomous aircraft capable of carrying various payloads, executing multiple tasks, and being reusable. Their unique features, such as cost-effectiveness, minimal losses, zero human casualties, high maneuverability, stealth capabilities, and adaptability, have significantly contributed to their widespread use in military, civilian, and scientific research fields^1–4^, thus fostering the growth of the drone industry.

The rapid expansion of the drone industry has resulted in the proliferation of numerous low-altitude UAVs. This surge has brought forth a range of challenges, particularly involving collision avoidance and countermeasures among drones. The notable emergence of these challenges emphasizes the urgency and significance of conducting air-to-air target detection within the drone domain^5–7^. In this context, achieving swift, precise, and reliable drone target detection remains pivotal in enhancing the safety and functionality of UAV systems^8–14^.

Various sensor types are available for UAV detection, including visual sensors^15–20^, radar^21,22^, and acoustic sensors^23,24^, among others. However, due to stringent payload constraints associated with UAVs, selecting the appropriate sensor is crucial. Visual sensors are preferred due to their lightweight nature, adaptability, and ability to provide high-quality image data. This study emphasizes the use of widely adopted RGB monocular cameras for UAV detection, offering promise for UAV detection applications and supporting future research and technological advancements.

In recent years, driven by the widespread utilization of artificial intelligence technology and the progress in object detection techniques rooted in deep learning and computer vision,object detection methods leveraging Convolutional Neural Networks (CNN) have shown superior performance in detection and recognition over traditional machine learning methods, prompting many CNN-based detection methods to be proposed. Currently, Computer vision-based object detection algorithms can be categorized primarily into two groups: the first group comprises two-stage object detection models exemplified by Faster RCNN^25^ , while the second group encompasses one-stage object detection models exemplified by YOLO^26,27^ and SSD^28^. Two-stage object detection models can effectively enhance detection accuracy through the utilization of region proposal networks for generating candidate object boxes.But the redundancy of structure and parameters makes it difficult to achieve fast detection. For one-stage object detection models,the primary advantage lies in its independence from the need for a region proposal network. and after training the backbone feature extraction network, it directly performs classification and regression on the input data, which can effectively shorten the training and inference time.

However, in the context of the widespread application of deep learning-based object detection technology, an increasing number of researchers have noted the issue of high computational demands, even within one-stage object detection models. This challenge results in these algorithms processing images acquired by computers at slower speeds, subsequently impacting their ability to achieve real-time object detection. Consequently, in recent years, some lightweight optimization approaches based on one-stage object detection models have been proposed with the aim of enhancing the efficiency of object detection. For example, Long et al.^29^ introduced LiraNet, a lightweight network tailored for ship detection in radar images. LiraNet amalgamates the concepts of dense connections, residual connections, and group convolution to enhance its efficacy.Furthermore, the researchers introduced a lightweight model, named Lira-YOLO, specifically designed for ship detection. This model employs LiraNet as its backbone network and incorporates a two-layer YOLO prediction layer for the purpose of object detection. Wang et al.^30^ proposed an efficient YOLO framework to address the limitations of traditional YOLOv3 in terms of model size and computation amount for on-road object detection tasks. The proposed framework achieves high accuracy while maintaining efficiency through iterative initialization strategy and comprehensive pruning schemes. Jiang et al.^31^ proposed a ship detection algorithm for Synthetic Aperture Radar (SAR) images using a multi-channel fusion method and the YOLO-V4-light deep learning framework. The proposed algorithm leverages image information and the network’s feature extraction capability, leading to a reduction in model complexity and detection time. The YOLO-V4-light network is optimized for three-channel images to mitigate the accuracy decline attributed to lightweighting.

The previously mentioned models have demonstrated outstanding performance in certain scenarios. Nonetheless, their effectiveness on embedded devices is limited due to the computational constraints of UAV onboard computing systems. Moreover, these models face hurdles in detecting air-to-air UAV targets due to various factors such as intricate image backgrounds, image clarity concerns, and the small size of target UAVs. Consequently, additional exploration is crucial to achieve superior air-to-air UAV detection performance while upholding lightweight characteristics.

In pursuit of efficient and precise air-to-air UAV detection, we developed YOLOv5s-ngn, utilizing the YOLOv5s backbone structure. Acknowledged for its outstanding real-time object recognition capabilities in images or videos, the YOLOv5s model underwent extensive validation in practical scenarios. Our methodology incorporates an inventive lightweight feature extraction network, employing channel splitting, channel reordering, and feature reuse concepts. This network optimizes the feature extraction process, supplanting the original YOLOv5s feature extraction network. Furthermore, we introduced a novel fusion module grounded on the Convolutional Block Attention Module (CBAM), which holistically models the feature pyramid structure of the backbone network by integrating spatial and channel attention mechanisms. This fusion unifies high-level semantic information and channel details while making predictions on a single feature layer, enhancing detection precision and curtailing model complexity. Additionally, we replaced the initial Complete IoU (CIoU) loss function with the Enhanced IoU (EIoU) loss function to accelerate convergence and refine regression accuracy, ultimately elevating the model’s overall precision.

The main contributions of this study include:

1. We have established a novel dataset for air-to-air unmanned aerial vehicle (UAV) target detection, taking into account the complexity of backgrounds in air-to-air scenarios. During the data collection process, we introduced various backgrounds, including residential areas, streets, fields, lakes, and mountainous terrain. Furthermore, the data collection efforts spanned the entire day, divided into three time periods: morning, noon, and evening, to ensure nearly equal data distribution across each time segment.
2. We have introduced a lightweight target detection model (YOLOv5s-ngn) by incorporating novel lightweight feature extraction modules, lightweight feature fusion modules, and introducing the EIoU function. This model achieves an improvement in model accuracy while maintaining its lightweight design.
3. We conducted extensive experiments to assess the effectiveness of our approach. The experimental results demonstrate that our model exhibits industry-leading performance on the UAVfly and Det-Fly datasets. Simultaneously, we deployed the trained model onto an unmanned aerial vehicle equipped with TX2 embedded hardware for real-time model detection. The experimental outcomes reveal that YOLOv5s-ngn can efficiently and swiftly detect unmanned aerial vehicles, enabling real-time application owing to its rapid response time.

The paper’s subsequent sections are arranged as follows: “Related work” provides an exploration of relevant research in the domain of target detection. Section “UAVfly dataset” provides a detailed description of the construction process of the UAVfly dataset. Section “Methods” delineates the specifics of the proposed methodology, and “Results” encompasses the experimental results. Lastly, the conclusion is presented in “Discussion”.

## Related work

### Research on unmanned aerial vehicle (UAV) detection

The field of UAV detection encompasses two primary scenarios: ground-to-air^21–24^ and air-to-air detection^32^, each with its own unique features and applications. Ground-to-air detection involves monitoring UAVs in flight from terrestrial vantage points, often requiring the deployment of monitoring equipment such as radar or visual cameras on the ground or stationary platforms. This method finds applications across various domains, including military air defense surveillance and civilian aviation air traffic management.Conversely, air-to-air detection involves UAVs using their onboard cameras or sensors to detect other UAVs in flight. This is critical for swift and accurate detection to avoid collisions and enable cooperative operations, especially in multi-UAV systems.

In recent years, research on ground-to-air UAV detection has gained significant attention due to the rising utilization of UAVs. However, air-to-air UAV detection poses even greater challenges that remain underexplored. A key reason for this complexity is the contrasting monitoring environments.

In many ground-to-air UAV detection scenarios, stationary or minimally mobile ground-based cameras^15–20^ capture images under relatively stable atmospheric conditions, such as clear or cloudy skies. Detection in such settings is comparatively straightforward due to the static background. Conversely, air-to-air UAV detection involves capturing images of UAVs in flight within dynamic and complex backgrounds, including urban and natural settings . These scenarios introduce additional visual complexities, as background elements, ground structures, and architectural features can obscure UAVs, exacerbating detection challenges. Furthermore, the dynamic flight characteristics of onboard cameras can lead to significant variations in the appearance of UAVs, affecting their shape, size, proportions, and color. This visual variability intensifies the difficulty of precise detection across diverse contexts.Another complicating factor is the small size of micro UAVs, making them inconspicuous in airspace and further increasing detection complexities. Effective solutions to air-to-air UAV detection necessitate addressing these challenges posed by diversity, complexity, and small-scale characteristics, representing a critical research focus in the current landscape.

### Methods for unmanned aerial vehicle (UAV) detection

Unmanned aerial vehicle (UAV) detection involves employing various methodologies rooted in sensor technology and signal processing:

- Radar Systems: These systems detect UAV-emitted radar signals, ensuring long-range detection reliability, even amidst varying environmental conditions.
- Infrared Sensors: Utilizing infrared radiation, these sensors capture thermal emissions from UAVs.
- Sonar Systems: Primarily intended for underwater UAVs, sonar technology is occasionally adapted for detecting aerial UAVs using sound waves.
- Radio Frequency Spectrum Analysis: This method identifies UAV communication signals, uncovering interactions between the UAV and its controller, facilitating inferences about the UAV’s position and operator.
- Image Processing and Machine Learning: Employing computer vision and machine learning algorithms, analysts assess aerial images and videos for UAV detection. These methods rely on discerning UAV visual features and movement patterns.

However, sensors such as radar, sonar, and radio frequency spectrum can suffer from interference from other UAV onboard sensors, potentially compromising accuracy. Additionally, most sensors lack the ability to identify specific target objects. Recent strides in computer vision have introduced vision-based techniques as innovative detection methods. Vision sensors operate in more relaxed experimental settings, boast lower production costs, and encompass attributes like extensive data collection and broad detection capabilities. Consequently, researchers are increasingly focusing on vision-based target detection techniques.

### Vision-based object detection methods

#### Traditional approaches

Visual object detection is the process of identifying, recognizing, and labeling specific objects in images, which is closely related to object classification, tracking, and image segmentation. Traditional object detection methods^33–35^ typically employ a sliding window strategy to scan the entire image with a series of sliding windows to determine possible object locations. Hand-crafted features, such as scale-invariant feature transform^36^ and histogram of oriented gradients^37^, are then extracted from the image window, followed by classification using support vector machine (SVM) or AdaBoost classifiers.However, traditional object detection algorithms based on the sliding window strategy have issues of high computational complexity, limited efficiency in detecting objects, and difficulty in handling changes in object shape and background. Additionally, designing hand-crafted features for each new object class requires considerable time.

Current research on UAV detection employs two fundamental technical approaches. Firstly, one method utilizes feature extraction techniques to capture UAV characteristics within images, such as shape, color, and texture. Subsequently, discriminative classifiers, such as Support Vector Machines (SVM) or Convolutional Neural Networks (CNN), are employed to analyze and categorize these extracted features, thereby determining the UAV’s position, category, or state^38,39^.

The second approach revolves around the detection of moving objects within images, with UAVs typically categorized as one type of moving object. Subsequently, generative classifiers are deployed to assess whether these moving objects correspond to UAV targets. This method emphasizes the analysis of trajectories, velocities, and motion patterns of moving objects to distinguish UAVs from other mobile entities^40–42^.

#### Deep learning-based approach

The domain of target detection methodologies in the realm of deep learning encompasses a diverse array of techniques and architectures. While Convolutional Neural Networks (CNNs) remain predominant, alternative methodologies persist. The subsequent elucidation presents some prominent deep learning-based target detection methods:

- Convolutional Neural Network (CNN) Methods: R-CNN Series: Encompassing R-CNN^43^, Fast R-CNN^44^, and Faster R-CNN^25^, these methodologies achieve target detection by introducing candidate regions and Region Proposal Networks (RPN). YOLO Series: Including YOLO (You Only Look Once)^45^, YOLOv2^46^, YOLOv3^26^, YOLOv4^27^, and YOLOv5^47^, these approaches treat target detection as a regression problem, enabling real-time detection. SSD (Single Shot MultiBox Detector)^28^: Efficient single-stage object detection is attained by concurrently detecting targets at different hierarchical levels and producing multiple detection results. RetinaNet^48^: Addressing the imbalance between positive and negative samples through Focal Loss, RetinaNet enhances detection performance for small targets while maintaining high recall.
- Non-Convolutional Approaches: Non-Convolutional Methods in Deep Learning: In addition to CNNs, certain deep learning-based target detection methods adopt non-convolutional structures, such as those based on Recurrent Neural Networks (RNNs)^49^ or attention mechanisms.
- Graph Neural Network (GNN) Approaches: Applications of GNN in Target Detection: Certain studies explore the application of Graph Neural Networks (GNNs) in target detection, leveraging the capture of relational information within graph structures to enhance detection performance^50^.
- Transformer-Based Approaches: Target Detection Based on Transformers: Recently, some endeavors have sought to apply Transformer architectures to the field of target detection, incorporating self-attention mechanisms to capture global and local relationships^51,52^.

These methodologies exhibit distinctive advantages and applicability in various application scenarios and tasks. Researchers continually explore novel deep learning approaches to address challenges in target detection, thereby expanding the developmental landscape of deep learning-based target detection technologies.

## UAVfly dataset

Datasets are indispensable for training object detection models, providing essential information to facilitate target learning. Their quality significantly influences model performance and generalization capabilities. Therefore, constructing datasets with high quality, diversity, representativeness, and balance is crucial for training and evaluating object detection models. However, collecting datasets for visually detecting air-to-air unmanned aerial vehicles (UAVs) is challenging due to the inherent complexities, such as complex backgrounds and image distortions from dynamic flights.

Currently, datasets explicitly designed for the precise detection of unmanned aerial vehicle (UAV) targets in the empty-to-empty scenario are exceedingly rare. Zheng et al.^32^. introduced a dataset named “Det-Fly” to address this gap. The dataset comprises 13,271 images of UAVs in the empty-to-empty scenario, captured from three different angles across four environmental backgrounds. Notably, it incorporates challenging backgrounds such as varying lighting conditions, dynamic blurring, and other factors that faithfully reflect the authentic operational processes of UAVs. However, practical utilization of models trained on this dataset reveals suboptimal accuracy in detecting UAVs in complex background settings. To address this issue, we propose a novel dataset named “UAVfly.” Its primary distinction from Det-Fly lies in providing more intricate empty-to-empty scenario images, thereby enhancing the dataset’s generalization capabilities. Table 1 illustrates the comparison between Dataset Det-Fly and Dataset UAVfly.

**Table 1:** Comparison between dataset det-fly and dataset UAVfly.

Model	Categories of backgrounds	Relative viewing angles	Proportion of small targets	Challenging scenarios
Det-fly	Sky urban field mountain	Front view (36.4%) Top view (32.5%) Bottom view (31.1%)	Approximately half	Strong/weak lighting (10.8%) Motion blur (11.2%) Partial target occlusion (0.8%)
UAVfly	Urban block Suburb Desert Field Lake Sky Mountain	Front view (33.8%) Top view (34.6%) Bottom view (31.6%)	30.2%	Strong/weak lighting (14.5%) Motion blur (13.8%) Partial target occlusion (1.1%)

In this study, we employed three unmanned aerial vehicle devices (DJI AIR2s) to collect datasets in an air-to-air fashion, conducted across three distinct Chinese provinces: Shanxi, Sichuan, and Guangdong.

The dataset consists of 10,281 images with a resolution of $1280 \times 720$ pixels. It comprehensively covers diverse geographical environments, encompassing urban blocks, suburbs, deserts, fields, lakes, skies, and mountains. Each environmental background type contributes nearly equally to the entire dataset, ensuring a high degree of diversity and representativeness. The dataset collection process spans an entire day, segmented into three time periods: morning, noon, and evening, with each period contributing almost equally to the dataset. This diverse and uniformly distributed data collection methodology ensures the dataset’s comprehensiveness and applicability, offering researchers a wealth of experimental material.

Regarding challenging backgrounds, we adopted the collection strategy from Dataset Det-Fly, accounting for factors such as varying lighting conditions, dynamic blurring, and partial occlusion of target objects. Specific efforts were not made to collect images of small target objects (objects with a height and width less than 10% of the entire image). Instead, images were captured at fixed time intervals during the UAV’s operation, ensuring coverage of various UAV horizontal distance scenarios in the dataset collection.

During the data collection process, we adhered to the following data collection strategies.

1. Throughout the data collection process, strict adherence to local regulations governing unmanned aerial vehicle (UAV) operations was maintained to ensure full compliance and safety.
2. In order to render the dataset versatile for a broad spectrum of applications within the low-altitude airspace domain, a comprehensive evaluation of background complexity was undertaken, leading to the establishment of a maximum UAV flight altitude of 100 m. Additionally, a mandatory constraint was enforced, stipulating that UAVs must maintain a minimum separation distance of 5 m, thereby guaranteeing the safe execution of data collection activities.
3. Within the dataset, a subset of images was captured by onboard monocular cameras at regular 0.5-s intervals. This data acquisition strategy proved advantageous in capturing temporal dynamics, including positional and state alterations of the targets at distinct timepoints, thereby imbuing the dataset with valuable temporal context.
4. Furthermore, to ensure data accuracy and usability, professional annotation software, LabelMe, was employed, and the annotation process was conducted by highly skilled experts. This meticulous annotation procedure facilitated the creation of a repository of meticulously annotated high-quality data, thereby establishing a reliable cornerstone for subsequent research endeavors.

**Figure 1:**
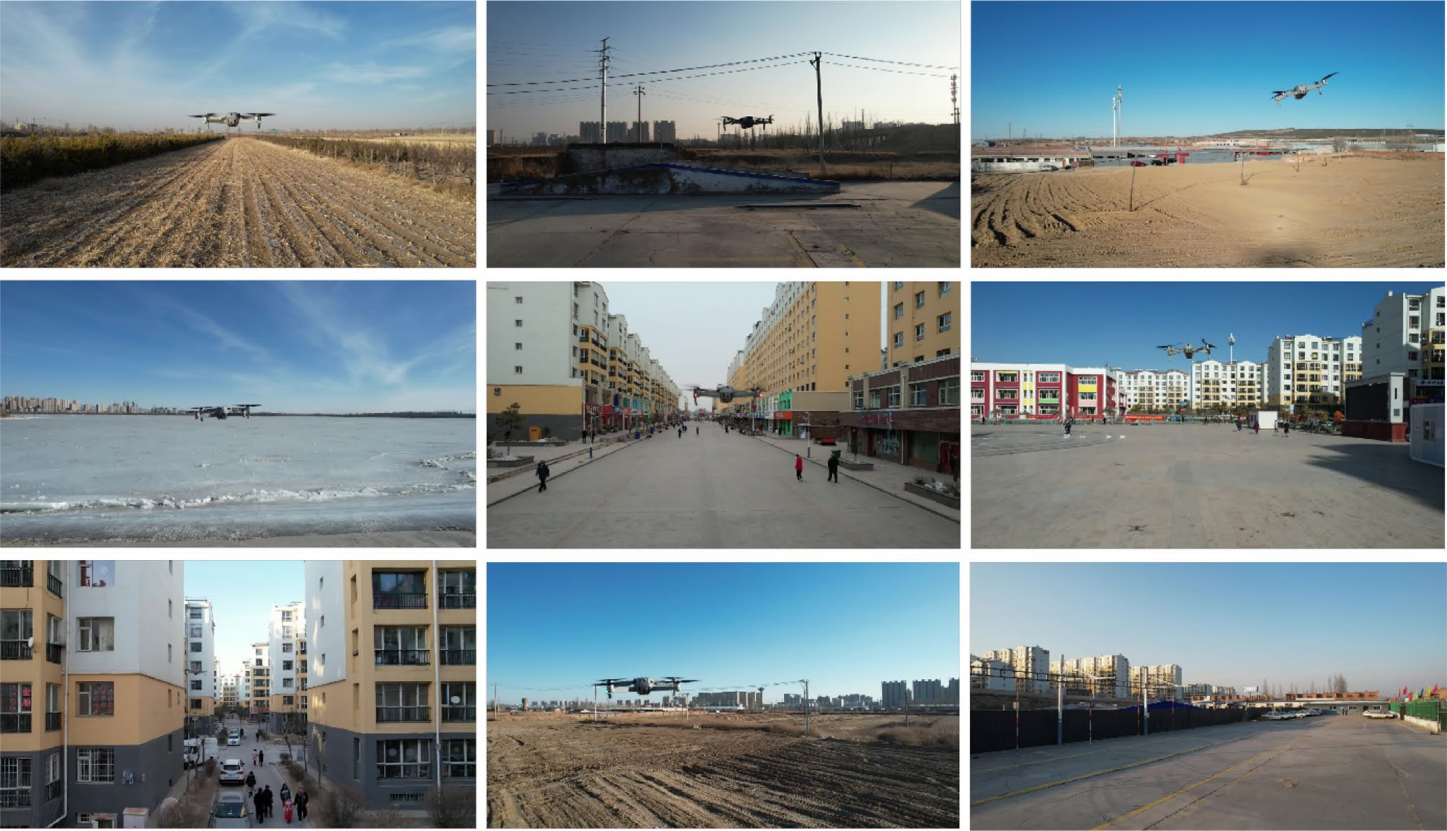
UAVfly dataset.

The self-constructed dataset is illustrated in Fig. 1. The dataset was partitioned randomly in a 7:3 ratio, leading to the creation of training and validation sets. This proportional division was orchestrated to maximize the effective use of the dataset for model training. Subsequent evaluation and validation were performed on the dedicated validation set to improve the model’s generalization performance. The employment of this random partitioning methodology serves to uphold dataset diversity and proficiently mitigate the risk of model overfitting to specific data distributions.

## Methods

### Abbreviation

As depicted in Table 2, explanations for the abbreviations referenced in the article have been provided.

**Table 2:** Abbreviation explanation.

CNN	Convolutional neural network
CBAM	Convolutional block attention module
ESA	Efficient spatial attention structures
FC	Fully connected
FPN	Feature pyramid network
GAP	Global average pooling
GAS	Global average squeezing
GMS	Global maximum squeezing
IoU	Intersection over union
PAN	Pyramid attention network
SGD	Stochastic gradient descent
SPP	Spatial pyramid pooling
SVM	Support vector machine

### The original YOLOv5s network

YOLOv5s is a highly efficient object detection model and the smallest in scale in the YOLOv5 series^47^. The model uses a lightweight backbone network and a multi-scale feature fusion and efficient output prediction strategy to achieve efficient, accurate, and real-time object detection^53,54^.

The YOLOv5s network architecture comprises an input section, backbone network, feature fusion module, and output section. The input segment employs the Mosaic-4 data augmentation technique, which enhances image information by utilizing cropping, concatenation, and resizing operations. This method is particularly effective for small object detection. The backbone network uses CSPDarknet53 as the feature extractor, which can improve feature extraction capabilities while maintaining efficiency.The feature fusion module employs a hybrid of Feature Pyramid Network (FPN) and Pyramid Attention Network (PAN) architectures to enhance the features extracted by the backbone network, thereby enhancing the network’s feature fusion capacity. The output end uses an efficient prediction strategy, including the use of multi-scale feature maps in the backbone network, feature fusion in convolution layers, and efficient processing in the output layer to achieve fast and accurate object detection.

**Figure 2:**
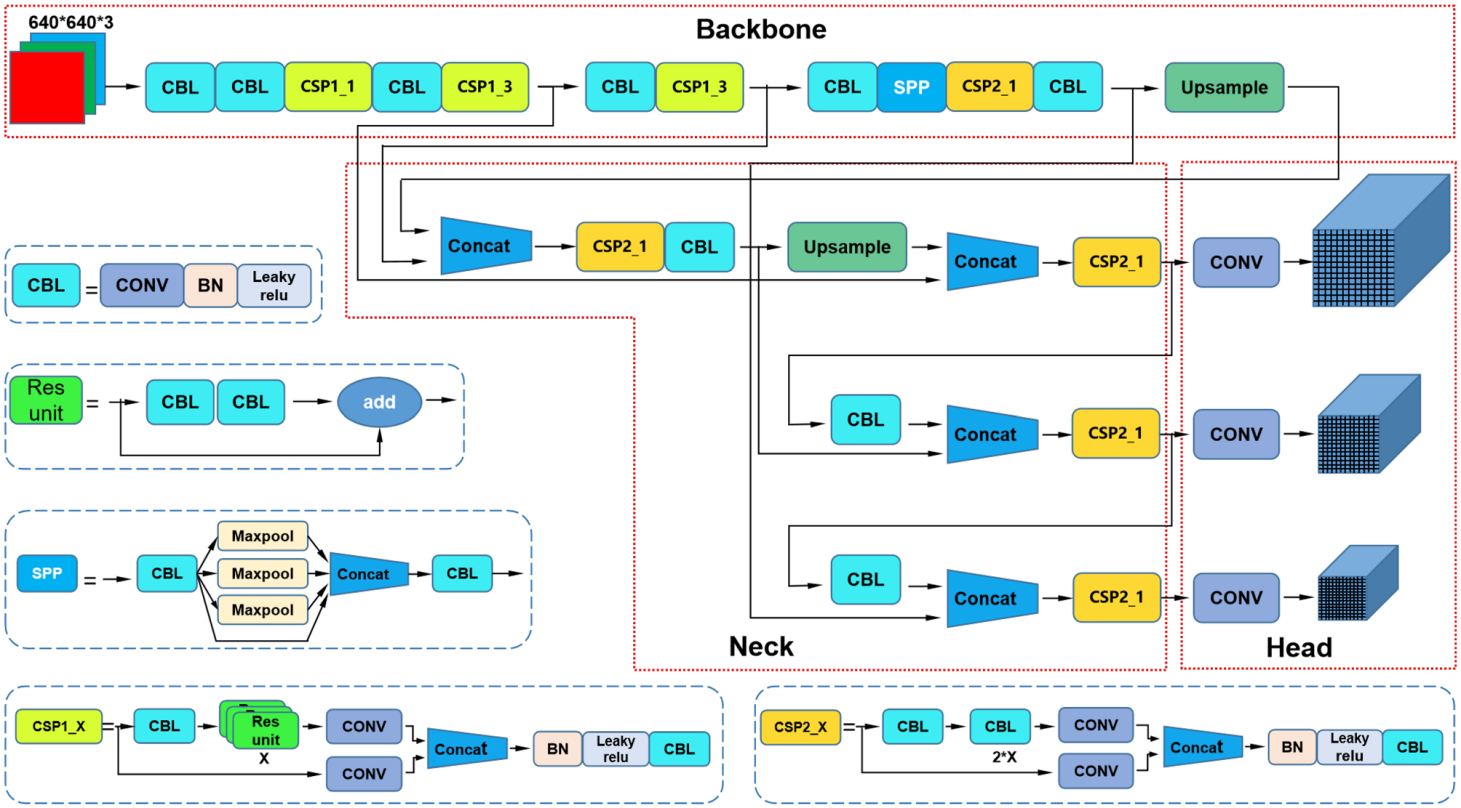
YOLOV5s structure.

Compared with other models in the YOLOv5 series, YOLOv5s has a smaller model size and faster inference speed but slightly lower accuracy than other larger models. The model demonstrates strong performance across various public object detection datasets and has found extensive utilization in diverse real-world applications, including intelligent surveillance, autonomous driving, and robotic vision. The YOLOv5s structure is illustrated in Fig. 2.

### Lightweight feature extraction

The feature extraction network in this paper has been improved in two parts: Firstly, the modification involves substituting the C3 module within the backbone network with CF2. Secondly, the alteration encompasses the transformation of the downsampling segment following the C3 module in the backbone network to MC.

**Figure 3:**
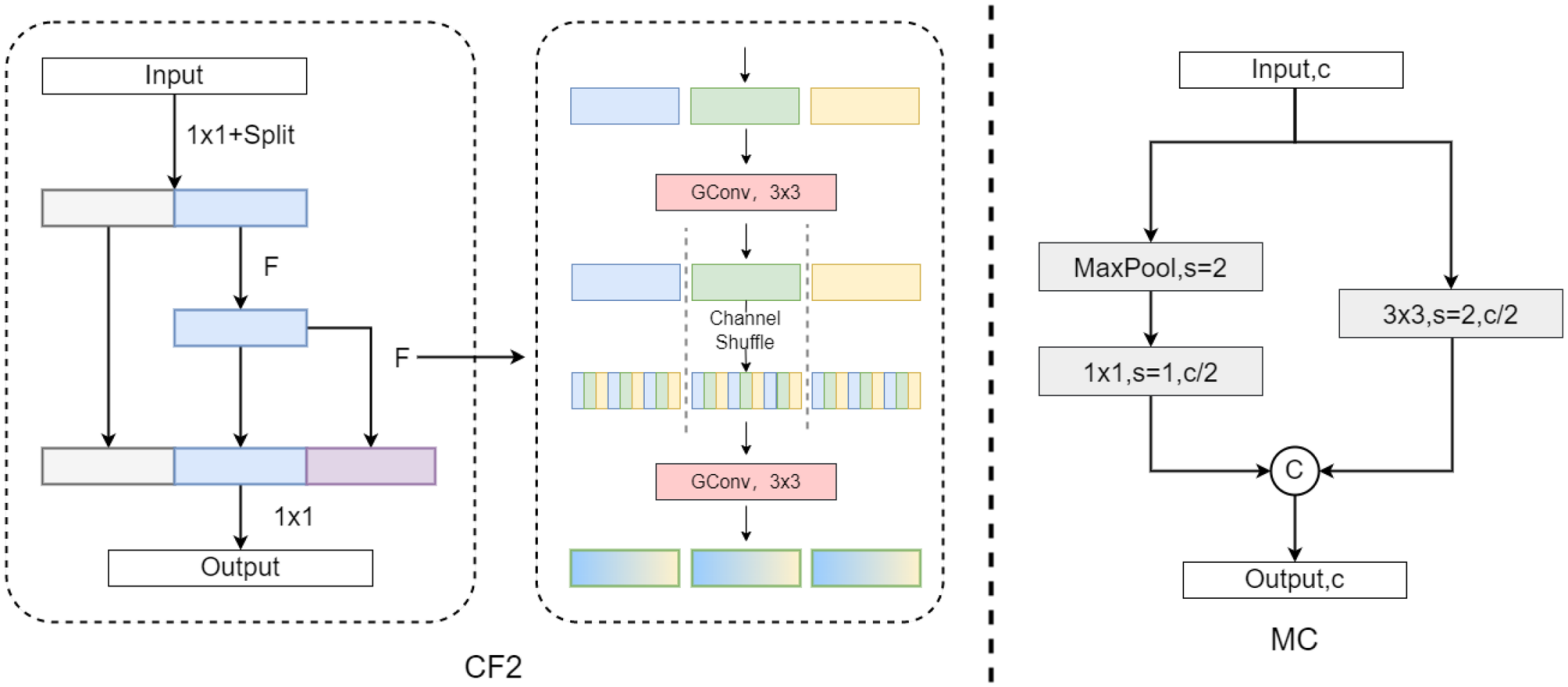
Lightweight feature extraction.

The CF2 network structure, illustrated in Fig. 3, processes the input by applying a $1 \times 1$ convolution and channel split operation to divide it into two sub-features (referred to as gray and blue features). In convolutional operations, channel splitting involves segmenting the input channels into multiple subsets, each subset undergoing convolution independently. This approach enhances network parallelism, thereby improving the computational efficiency of the model and enabling more effective utilization of hardware resources within the network.

Sub-feature 1 (gray) remains unchanged, while sub-feature 2 (blue) is sent to the F module for feature learning. The learned blue feature is then fed into the F module again to generate the purple feature. The gray, blue, and purple multi-scale features are concatenated to enhance feature reuse, and are then sent to a $1 \times 1$ convolution to obtain the output feature. The idea of multi-scale feature concatenation and feature reuse comes from VOVNet^55^. Feature reuse refers to the process of utilizing features extracted from preceding layers and employing them in subsequent layers. In neural networks, lower-level features usually encompass more fundamental information, while higher-level features encapsulate more abstract and advanced characteristics. By enabling the model to efficiently leverage information gleaned from earlier layers, feature reuse enhances the model’s performance and efficiency. This technique has the potential to diminish computational complexity, decrease the number of parameters, and shorten training time, all while enhancing the model’s generalization capabilities.

The F module consists of two $3 \times 3$ grouped convolutions, where channel shuffling is performed to facilitate inter-group feature interactions^56^. The channel shuffle technique is frequently employed on the output of convolutional layers that possess multiple channels. Its purpose is to facilitate the exchange and fusion of information among channels by reordering the arrangement of channels, thereby amplifying the network’s representational capacity. The channel shuffle operation encompasses grouping, interlacing, and reconfiguring feature maps derived from various channels. This procedure helps enhance the model’s capability to abstractly represent features, thereby reinforcing interrelations and diversification among features, ultimately contributing to optimizing the model’s performance.

The MC network structure, as shown in the Fig. 3, is mainly inspired by the downsampling method in PeleeNet^57^. The Stemblock structure in PeleeNet ensures strong feature expression ability and reduces a significant number of parameters. In the MC structure, one branch uses max pooling and a $1 \times 1$ convolution to reduce the number of channels by half (represented by c in the figure), while the other branch uses a $3 \times 3$ convolution with a stride of 2 to reduce the number of channels by half. The outputs of the two branches are then concatenated to obtain the output feature, ensuring that the final result has sufficient semantic information while reducing the number of parameters and avoiding excessive loss of information.

### Lightweight feature fusion

In the feature fusion section, this paper extensively utilizes spatial and channel attention mechanisms to holistically model the high-level semantic information and channel-wise characteristics derived from the feature pyramid structure of the backbone network^58^.

The spatial and channel attention mechanism is a technique employed in neural network architectures, notably in convolutional neural networks (CNNs), to refine feature representation.The spatial attention mechanism is geared toward identifying and accentuating pertinent spatial areas within an image. It allocates weights to distinct spatial locations, highlighting regions of greater significance for the given task. This approach enables the network to focus on vital image sections, thereby bolstering its capacity to effectively capture spatial information.Conversely, the channel attention mechanism strives to accentuate crucial channels within the feature maps generated by diverse network layers. It assesses interdependencies among channels and assigns weights to emphasize channels harboring more pertinent information for the designated task. This process empowers the network to prioritize and concentrate on informative channels, thus enhancing its ability to extract relevant features from the data.The coordinated interplay of spatial and channel attention mechanisms enables the network to better discern critical spatial areas and channel-specific features within the data.

**Figure 4:**
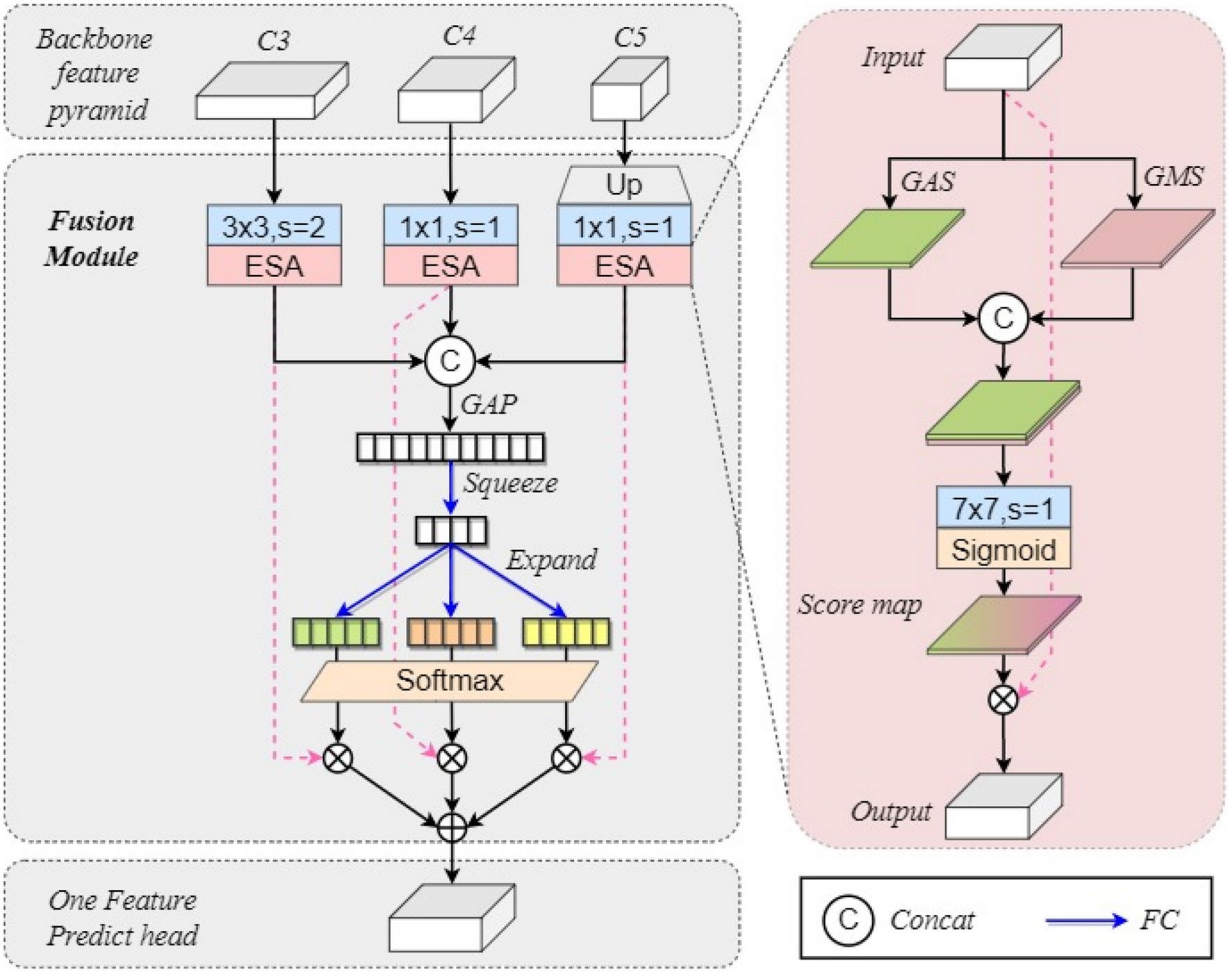
MG feature fusion module.

A novel fusion module (MG) is devised, and predictions are generated based on a distinct feature layer.The MG network structure, as shown in the Fig. 4.

To ensure alignment in terms of channel and feature map size, the feature pyramid structure C3, C4, C5 obtained from the backbone network undergoes operations such as convolution with a stride of 2, $1 \times 1$ convolution, and upsampling with a $1 \times 1$ convolution. Efficient spatial attention structures (ESA) are subsequently introduced after each convolutional structure. Within the ESA, channel information is effectively compressed by employing two global compression techniques along the channel dimension, namely global average squeezing (GAS) and global maximum squeezing (GMS). This compression process facilitates the formation of comprehensive global information in the spatial domain. The two branches of features are then concatenated and processed using a $7 \times 7$ convolutional operation, enabling effective fusion of the global information. The resulting score map, after sigmoid activation, is element-wise multiplied with the original feature map.

Moreover, the concatenated feature maps derived from the three layers undergo global average pooling (GAP), leading to the generation of a one-dimensional vector encapsulating the global channel-wise information for the multi-scale feature maps. This vector is subsequently subjected to global information compression through fully connected (FC) layers, a process referred to as “squeeze”^59^. Additionally, the squeezed vector is separately fed into three distinct FC layers, facilitating distinct learning of channel-wise information for the three feature maps. Consequently, three different vectors (designated as green, dark yellow, and light yellow) are obtained. By applying the softmax function to each vector, attention levels for the diverse multi-scale global information are effectively discerned. Following the softmax operation, the vectors are element-wise multiplied with their corresponding scale feature maps, and subsequently aggregated to yield the final single-feature prediction head.

### Improvement of the loss function

#### Intersection over union (IoU) loss

**Figure 5:**
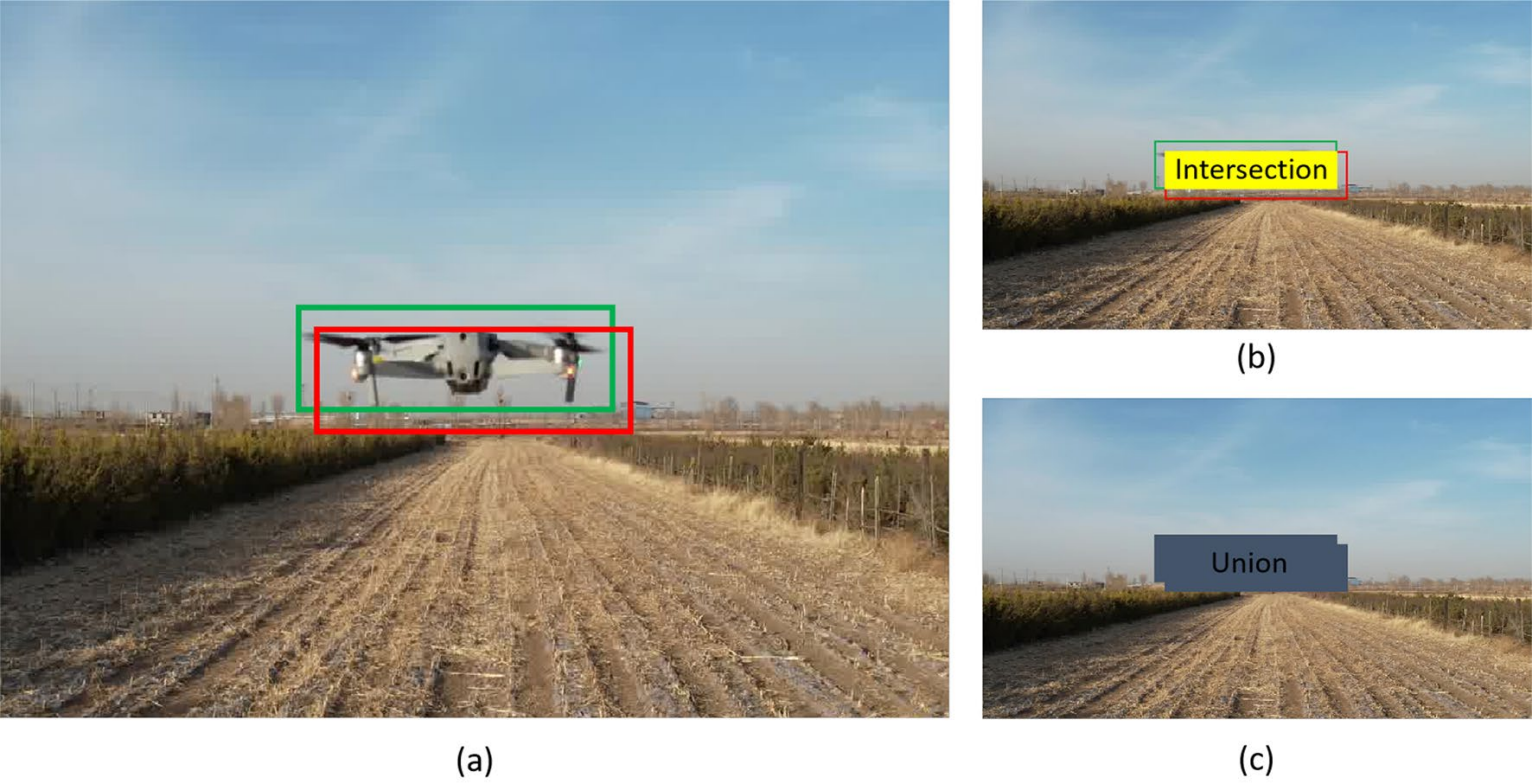
Intersection over Union (IoU) schematic diagram: (**a**) illustrates the positions of the predicted box and the ground truth box, (**b**) shows the intersection between the predicted box and the ground truth box, and (**c**) represents the union of the predicted box and the ground truth box.

Illustrated in Fig. 5, the red box signifies the predicted bounding box, while the green box represents the ground truth box (annotated box). The Intersection over Union (IOU) quantifies the extent of overlap between the ground truth box and the predicted box through the subsequent steps:

- Compute the intersection area between the ground truth box and the predicted box.
- Calculate the union area between the ground truth box and the predicted box.
- Determine the IOU ratio by dividing the intersection area by the union area.
  1 $$\begin{aligned} IoU= \frac{Intersection}{Union} \end{aligned}$$

The IoU Loss is defined as follows:

2 $$\begin{aligned} IoU Loss= 1-IoU \end{aligned}$$

The Intersection over Union (IoU) metric assesses the detection performance between predicted and ground truth bounding boxes. It possesses scale invariance, demonstrating insensitivity to scale variations. In regression tasks, IoU serves as a direct indicator of the distance between the predicted box and ground truth (GT). However, IoU encounters the following limitations:

- Inability to conduct gradient backpropagation: The IoU cannot be used directly for gradient updates. When IoU equals 0 (indicating no intersection between the two boxes), computing a loss as $1 - {\text {IoU}}$ leads to difficulties in gradient backpropagation.
- Incapability to ascertain the distance between predicted and ground truth boxes: IoU yields 0 when there is no intersection between the boxes, making it challenging to gauge the distance between these boxes.
- Lack of information about the nature of intersection between boxes: IoU fails to describe how the two boxes intersect or the overlap pattern they exhibit.
- Inability to precisely quantify the degree of overlap between two boxes: IoU lacks the granularity needed to precisely measure the level of overlap or coincidence between the two bounding boxes.

#### CIoU loss function

YOLOv5s utilize the CIoU Loss function, which is defined as follows:

3 $$\begin{aligned} L_{CIoU}=1-IoU+\frac{\rho ^{2}(b,b^{gt}) }{c^{2}}+\alpha v \end{aligned}$$

The values of $\alpha$ and v are defined as:

4 $$\begin{aligned}{} & {} v=\frac{4}{\pi ^{2}}(arctan\frac{w^{gt}}{h^{gt}}-arctan\frac{w}{h})^{2} \end{aligned}$$

5 $$\begin{aligned}{} & {} \quad \alpha =\frac{v}{\left( 1-IoU\right) +v} \end{aligned}$$

The gradient of the CIoU Loss function with respect to the side lengths w and h is:

6 $$\begin{aligned} \left\{ \begin{array}{ll} \frac{\delta _{v}}{\delta _{w}}=\frac{8}{\pi ^{2}}\left( arctan\frac{w^{gt}}{h^{gt}}-arctan\frac{w}{h}\right) \times \frac{h}{w^{2}+h^{2}}\\ \frac{\delta _{v}}{\delta _{h}}=-\frac{8}{\pi ^{2}}\left( arctan\frac{w^{gt}}{h^{gt}}-arctan\frac{w}{h}\right) \times \frac{w}{w^{2}+h^{2}} \end{array}\right. \end{aligned}$$

In the equation, b and $b^{gt}$ correspond to the centers of the predicted box and the ground truth box, respectively. The term $\rho ^{2}$ indicates the Euclidean distance between these centers, while c represents the diagonal length of the smallest encompassing closed region that includes both the predicted and ground truth boxes.

While the CIoU Loss function incorporates considerations for bounding box regression, including overlap area, center point distance, and aspect ratio, the inclusion of the penalty term for relative proportion in the CIoU Loss function does not affect the regression process when the width and height aspect ratios of the predicted and ground truth boxes exhibit a linear relationship. Additionally, the gradient values of w and h have opposite signs, meaning that an increase in one value results in a decrease in the other value, making it impossible to keep them increasing or decreasing simultaneously.

#### EIoU Loss function

The EIoU Loss function represents an enhanced iteration of the CIoU Loss function^60^. In contrast to the CIoU Loss function, the EIoU Loss function employs an EIoU metric to quantify the overlap between the predicted and ground truth bounding boxes. It directly imposes penalties on the predicted width and height results.The EIoU metric effectively penalizes discrepancies in the predicted width and height from the ground truth values. Furthermore, it circumvents the gradient conflict issue encountered during gradient computation in the CIoU Loss function.The EIoU Loss function is defined as:

7 $$\begin{aligned} L_{EIoU}=L_{IoU}+L_{dis}+L_{asp}=1-IoU+\frac{\rho ^{2}\left( b,b^{gt}\right) }{c^{2}}+\frac{\rho ^{2}\left( w,w^{gt}\right) }{C_{w}^{2}}+\frac{\rho ^{2}\left( h,h^{gt}\right) }{C_{h}^{2}} \end{aligned}$$

The variables $C_{w}$ and $C_{h}$ represent the width and height of the predicted box and the ground truth box minimum bounding rectangle, respectively.The EIoU Loss function partitions the loss function into three components: overlap loss ($L_{IoU}$), center distance loss ($L_{dis}$), and width-height loss ($L_{asp}$). The aspect ratio loss term is separated into the discrepancy between the predicted width and the width of the minimum bounding rectangle, along with the discrepancy between the predicted height and the height of the minimum bounding rectangle.This approach enhances the aspect ratio loss convergence rate and enhances regression precision.

## Results

We conducted extensive model training on the UAVfly and Det-Fly^32^ datasets to comprehensively validate the effectiveness of our proposed algorithm. This meticulous training process was undertaken with the aim of ensuring the algorithm’s robustness and generalization capabilities across diverse datasets, further substantiating its outstanding performance in the field of unmanned aerial vehicle (UAV) visual detection. The combined utilization of these two datasets facilitated the capture of UAV images in various scenarios and contexts, enabling a more comprehensive assessment of the algorithm’s performance and reliability.

### Experimental environment

The training environment for all models in this experiment was Windows 10, with an AMD Ryzen 5 4600H with Radeon Graphics 3.00 GHz CPU, 16 GB of RAM, an NVIDIA GeForce GTX 1660 Ti GPU, and Pytorch as the deep learning framework. The Python version used was 3.7.

### Datasets

- UAVfly. The detailed information regarding the dataset UAVfly is elaborated upon in “UAVfly dataset”. We collected datasets in air-to-air form using three unmanned aerial vehicle devices (DJI AIR2s) .The datasets encompass residential areas, streets, fields, lakes, and mountains. Each environmental background type contributes nearly equally to the overall dataset.
- Det-Fly. The Det-Fly dataset^32^ comprises over 13,000 images of airborne unmanned aerial vehicles taken by a DJI Mavic2 drone. It encompasses diverse real-world scenarios, featuring varied background scenes, viewing angles, relative distances, and flying altitudes. The dataset contains data from three different perspectives, including front view , top view , and bottom view .

### Evaluation metrics

#### The evaluation metrics for lightweightness

This paper employs three key metrics to evaluate the lightweight nature of the model: the number of parameters required (Param), floating-point operations (FLOP), and Frames Per Second (FPS). The number of parameters represents the total model parameter count affecting memory usage and program initialization time. The parameter count is specific to the network model, and once confirmed, it remains unchanged. During model lightweighting, the parameter count decreases. Upon completion of model training, each parameter has a precise value, allowing direct usage of parameter files for target predictions during detection tasks.

Floating-point operations (FLOP) serve as a metric to measure algorithmic complexity and are commonly used as an indirect measure of the speed of neural network models. Each multiplication or addition represents one FLOP. The computational complexity (FLOP) indicates the model’s demand on hardware computational units and reflects the number of multiplications and additions required for forward inference.

Frames Per Second (FPS) refers to the number of frames (images) the network can process (detect) per second. It evaluates the detection speed, depicting the quantity of images processed per second or the time needed to process a single image. A shorter time implies a faster speed. FPS serves as a direct measure of the neural network model’s speed, validating the algorithm’s detection speed in subsequent embedded experiments.

#### The performance evaluation metrics of the model

In machine learning and deep learning, the prediction outcomes of a classification task are categorized into the following four types, collectively known as the confusion matrix:

- True Positive (TP): Predicted positive and labeled positive, indicating a correct prediction.
- False Negative (FN): Predicted negative but labeled positive, indicating a misclassification.
- False Positive (FP): Predicted positive but labeled negative, indicating a misclassification.
- True Negative (TN): Predicted negative and labeled negative, indicating a correct prediction.

Here, positive and negative refer specifically to unmanned aerial vehicles.

Object detection algorithms typically use several metrics to evaluate their performance, including precision, recall, average precision (AP), and mean average precision (mAP). The equations for calculating precision, recall, AP, and mAP are as follows:

8 $$\begin{aligned} Precision = \frac{TP}{TP+FP} \end{aligned}$$

Upon analysis of the formula, it is evident that Precision concerns the predicted positives in relation to the true positives and negatives. As Precision increases, the number of False Positives (FP) diminishes. This reduction signifies fewer instances of misclassifying other categories as the designated class, indicating higher purity in the predicted positives. Higher Precision corresponds to fewer false alarms or instances of erroneous detections.

9 $$\begin{aligned} Recall = \frac{TP}{TP+FN} \end{aligned}$$

Upon analysis of the formula, it is apparent that Recall concerns the predicted positives and negatives in relation to the true positives. As Recall increases, the number of False Negatives (FN) decreases. This decrease implies fewer instances of misclassifying positives as negatives, indicating a higher capacity to capture a larger portion of all actual positives. Higher Recall corresponds to fewer instances of missed detections or lower rates of failing to identify actual positives.

10 $$\begin{aligned} AP=\int _{0}^{1}PdR \end{aligned}$$

Although named average precision (AP), the calculation method of AP does not involve computing the average of Precision values. Instead, it computes the area enclosed by the Precision-Recall curve and the coordinate axes for each class, utilizing integral methods for computation. If a model has a larger AP, signifying a larger area enclosed by the Precision-Recall curve and the coordinate axes, it implies higher Precision and Recall overall.

11 $$\begin{aligned} mAP=\frac{1}{n}\sum _{i=0}^{n}AP_{i} \end{aligned}$$

The mAP averages the AP values for all classes. AP reflects the precision of predictions for each class, while mAP represents the average AP across all classes, serving as an indicator of the overall accuracy of the entire model.

The mAP encompasses two forms: one is mAP@0.5, which denotes the mAP value at an IOU threshold of 0.5. In this scenario, when the Intersection over Union (IOU) between the predicted box and the annotated box exceeds 0.5, the object is considered predicted correctly. The mAP is then computed under this premise. The other form is mAP@[0.5:0.95], which represents the mAP across multiple IOU thresholds within the range [0.5, 0.95] with an interval of 0.05. It involves utilizing ten IOU thresholds within this range to compute their respective mAP values, followed by averaging these values. A larger mAP@[0.5:0.95] indicates more accurate predicted boxes as it encompasses a wider range of higher IOU thresholds.

### Training parameters

The YOLO algorithm is a widely used method for object detection, and the training parameters play a crucial role in determining the model’s effectiveness and precision. Here’s a succinct overview of key parameters and their respective functions:

- Number of Epochs: Specifies the number of complete passes the model makes through the entire dataset during training. Increasing epochs may improve model performance but can also lead to overfitting.
- Batch Size: Determines the quantity of samples fed into the model for weight updates in each iteration. Larger batch sizes generally expedite training but may require more memory resources.
- Learning Rate: Governs the size of adjustments made to model parameters during training. A higher learning rate can hasten convergence but might result in unstable training. Conversely, a lower learning rate could demand more training time but contributes to a more stable convergence towards an optimal model.
- Optimizer: An algorithm employed to fine-tune model weights to minimize the loss function. Common optimizers include Stochastic Gradient Descent (SGD), Adam, RMSprop, each offering distinct advantages and suitability in various contexts.
- Image Size: Specifies the dimensions of input images. Larger image sizes generally enhance detection accuracy but also increase computational load and training duration.

The meticulous selection and fine-tuning of these parameters can significantly impact training speed, model performance, and convergence. Strategic adjustments aid the model in better adapting to the dataset, ultimately enhancing detection accuracy.

**Figure 6:**
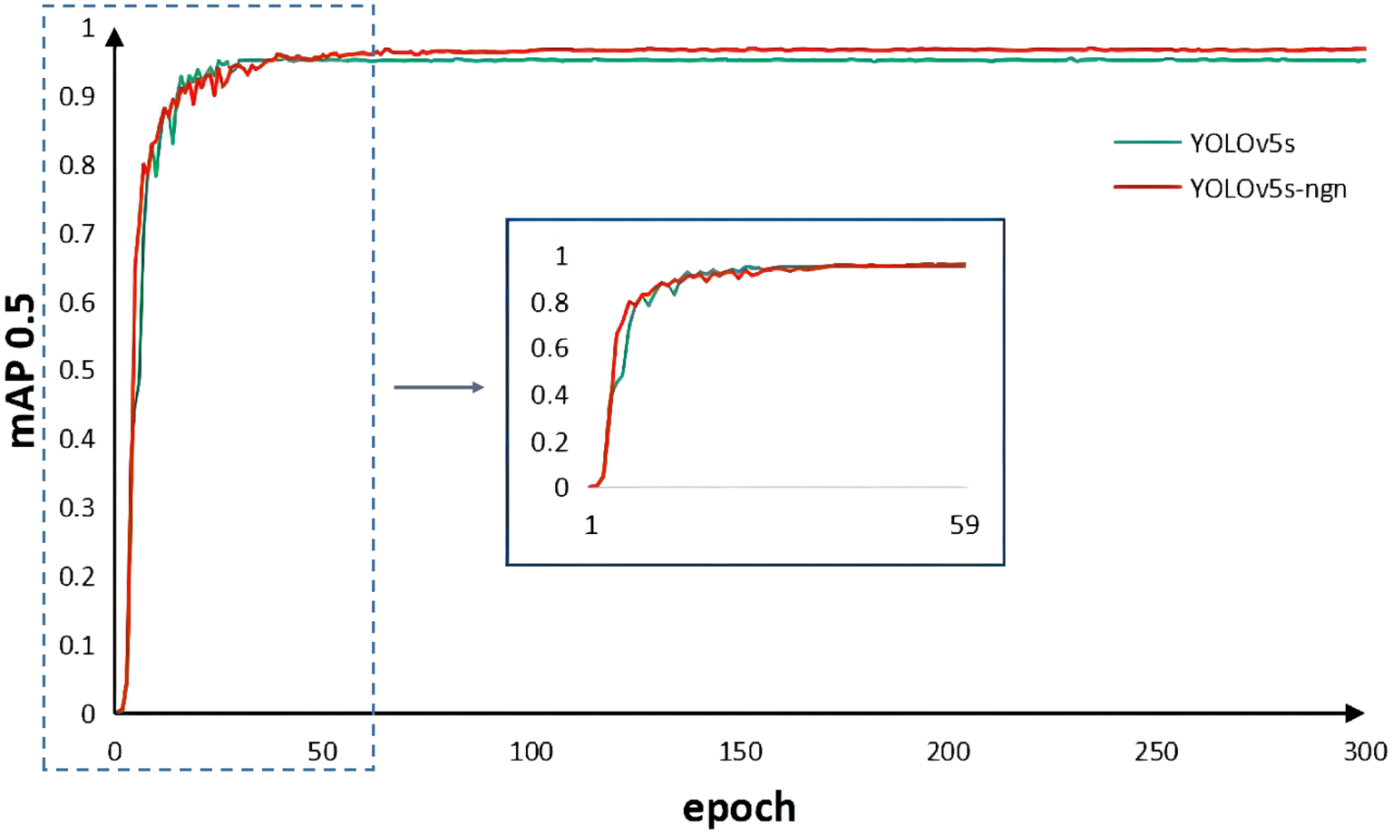
Comparison of mAP between the improved algorithm and the original algorithm during training.

The hyperparameter settings for this experiment are as follows: initially, experiments involved varying the training epochs between 100, 200, 300, and 400 to optimize model performance while minimizing overfitting risks. Ultimately, we determined the optimal training epochs as 300. Considering the influence of batch size on memory usage and guided by equipment limitations, a batch size of 16 was chosen for training. The training images were standardized to dimensions of $640 \times 640$ pixels. Stochastic Gradient Descent (SGD) served as the optimizer for this study. To dynamically regulate the learning rate during training, we initialized the lr0 (initial learning rate) at 0.01, applying the cosine annealing algorithm for adjustments. Figure 6 depicts the comparative analysis of mean Average Precision (mAP) between our modified algorithm and the original approach post-parameter tuning. The graphical representation distinctly exhibits the enhanced model’s superiority in both detection accuracy and convergence speed over the original YOLOv5s.

### Comparative experiments of YOLOv5s with different backbone networks

This section presents a comparative experiment on three different backbone networks of YOLOv5 using the UAVfly dataset to validate the effectiveness of improving the backbone network with CF2-MC-SPP. The experimental model details are as follows: First, the backbone network C3Net is replaced by our proposed lightweight convolutional neural network CF2-MC to obtain model 2. Then, the YOLOv5-CF2-MC backbone network is restructured by adding a Spatial Pyramid Pooling (SPP) module to obtain model 3. To validate whether the improved lightweight backbone model can reduce network complexity, decrease network computation, and effectively reduce inference time, this study conducted comparative experiments using unimproved YOLOv5s and two lightweight networks, Model 2 and 3. The evaluation metrics for the three different backbone networks of YOLOv5s are shown in Table 3.

**Table 3:** Comparative experiment of different backbone networks for YOLOv5s.

Model	Backbone network	Param M	FLOP G	mAP 0.5	mAP 0.5:0.9
1	YOLOv5s (baseline)	7.02	15.8	**0.936**	0.594
2	CF2-MC	**5.31**	**9.2**	0.921	0.577
3	${\text {CF2}}-{\text {MC}}+{\text {SPP}}$	5.65	10.0	0.935	**0.598**

Table 3 displays that model 2, replacing the lightweight convolutional neural network CF2-MC directly as the YOLOv5s backbone, exhibits a slightly reduced detection accuracy compared to the original YOLOv5s. However, its lightweight metric significantly outperforms the latter. The experimental results illustrate the efficacy of employing CF2-MC to achieve model lightweighting. The substantial reduction in parameter count and computational demands contributes to effectively lowering the hardware prerequisites for both model training and inference.To compensate for the accuracy loss caused by lightweighting, the CF2-MC backbone network was optimized by adding an SPP module after the last convolutional layer of the YOLOv5-CF2-MC backbone network, and the model’s mAP@0.5 increased from 92.16 to 93.5%. The outcomes indicate that employing the enhanced CF2-MC-SPP as the backbone network leads to a substantial enhancement in detection accuracy in comparison to model 2. Through this experiment, the effectiveness of the lightweight backbone model based on CF2-MC-SPP has been validated.

### Ablation experiments of feature fusion network models

Merely relying on the lightweight design of the backbone network is insufficient to meet our requirements. In this section, we conducted lightweight ablation experiments on the feature fusion part of YOLOv5s using the UAVfly dataset to achieve a lower parameter count and higher detection accuracy.

In the ablation experiments, YOLOv5s was used as the baseline. Model A was obtained by replacing the backbone network of YOLOv5s with CF2-MC-SPP. Building upon Model A, Model B was derived by introducing the MG fusion module, and Model C was developed by incorporating the EIoU function.

Table 4 presents the results of the ablation experiments, demonstrating that by replacing the backbone network of YOLOv5s with CF2-MC-SPP and utilizing both the MG fusion module and the EIoU function, the YOLOv5s-ngn model achieved a reduction in parameter count and an improvement in accuracy. Model B exhibited a 57.7% reduction in parameter count and a 41% reduction in FLOP compared to Model A, with a corresponding 0.7% increase in mAP0.5. Model C, which solely employed the EIoU function, showed no change in parameter count and FLOP compared to Model A, but achieved a 1.3% increase in mAP0.5. Subsequently, Model D (YOLOv5s-ngn) was derived by incorporating the EIoU function into Model B, further enhancing detection accuracy. These experiments validate the performance of the proposed improved model. YOLOv5s-ngn achieved a balance between speed and accuracy by reducing the parameter count, while surpassing the detection accuracy of the original YOLOv5s.

**Table 4:** Ablation experiments of YOLOv5s-ngn. (“$\checkmark$” indicates the usage of this model).

Model	Baseline	${\text {CF2}}-{\text {MC}}+{\text {SPP}}$	MG	EIoU	Param M	FLOP G	mAP 0.5	mAP 0.5:0.9
YOLOv5s	$\checkmark$				7.02	15.8	0.936	0.594
A		$\checkmark$			5.65	10.0	0.935	0.598
B		$\checkmark$	$\checkmark$		**2.39**	**5.9**	0.942	0.612
C		$\checkmark$		$\checkmark$	5.65	10.0	0.948	0.607
D		$\checkmark$	$\checkmark$	$\checkmark$	**2.39**	**5.9**	**0.954**	**0.615**

### Comparative experiments of classical object detection networks

#### Experimental results on the self-constructed UAVfly dataset

In order to confirm the superiority of the proposed YOLOv5s-ngn network over conventional object detection algorithms, we conducted training using various network models of object detection algorithms on a dataset we created. To ensure experimental reliability, YOLOv7^61^, YOLOX^62^, YOLOv5, YOLOv4^27^, and YOLOv3^26^ were trained using identical hyperparameters to their unimproved counterparts. The evaluation metrics for these eight network models are presented in Table 5.

**Table 5:** Comparative experiment of classic target detection network.

Model	Param M	FLOP G	Urban block	Suburb	Desert	Field	Lake	Sky	Mountain	mAP 0.5
YOLOv5s	7.02	15.8	0.887	0.912	0.948	0.944	**0.968**	0.971	0.921	0.936
YOLOv4	52.5	119.8	0.874	0.922	0.931	0.945	0.933	0.958	0.906	0.924
YOLOv3	61.15	192.1	0.821	0.889	0.912	0.918	0.921	0.920	0.884	0.895
YOLOv5m	20.9	48.2	0.884	0.917	0.947	0.951	0.954	0.969	**0.967**	0.941
YOLOX-s	8.94	26.7	0.872	**0.953**	0.959	0.968	0.951	0.972	0.958	0.949
YOLOv7	36.49	103.5	0.932	0.952	0.976	**0.976**	0.961	0.982	0.948	**0.961**
YOLOv7-tiny	6.01	13.2	0.901	0.948	0.952	0.959	0.952	0.971	0.953	0.948
YOLOv5s-ngn	**2.39**	**5.9**	**0.941**	0.951	0.954	0.961	0.967	**0.983**	0.923	0.954

Based on the detection results in Table 5 for the eight network models, it is evident that the YOLOv5s-ngn model outperforms all six models, except YOLOv7, in mAP for UAV object detection. Furthermore, it also surpasses the other seven models in lightweight metrics, including parameter count and FLOP.These findings underscore the exceptional performance of the YOLOv5s-ngn model in UAV object detection tasks. However, alternative approaches exhibit limited generality in this context, encountering challenges such as insufficient detection accuracy and slow detection speeds. Therefore, the choice of YOLOv5s-ngn as the target detection algorithm for identifying UAV objects is highly compelling. This selection not only ensures high detection accuracy but also provides significant advantages in lightweight design, offering robust support for real-time UAV object detection.

#### Experimental results on the Det-Fly dataset

We conducted a comprehensive comparison between YOLOv5s-ngn and mainstream algorithms applied to the Det-Fly dataset, with specific results presented in Table 6. These findings reveal that YOLOv5s-ngn achieves outstanding detection accuracy in urban and sky backgrounds. Additionally, its mean Average Precision (mean AP) surpasses that of the other eight algorithms significantly across the four different background conditions. This underscores the exceptional performance of YOLOv5s-ngn across various background environments, highlighting its prowess in target detection tasks.

**Table 6:** Comparative experiments of YOLOv5s-ngn with other mainstream methods on the Det-Fly Dataset.

Algorithms	Field	Urban	Sky	Mountain	mAP 0.5
Casade R-CNN^63^	68.1	67.4	94.5	**81.8**	77.950
FPN^64^	71.7	71.5	85.1	77.8	76.525
Faster R-CNN^25^	65.2	61.5	87.8	79.2	73.425
Grid R-CNN^65^	**76.2**	78.2	91.5	73.2	79.775
RefineDet^66^	69.4	55.8	87.4	74.1	71.675
RetinaNet^48^	74.4	71.6	89.8	71.2	76.750
SSD512^28^	75.1	68.8	93.1	77.8	78.700
YOLOv3^26^	68.8	61.2	87.5	79.2	74.175
YOLOv5s-ngn	74.8	**79.1**	**95.2**	79.8	82.225

### Embedded experiments

The NVIDIA TX2 is an embedded artificial intelligence computing platform designed by NVIDIA. It incorporates a high-performance NVIDIA Pascal architecture GPU, providing robust computational power and energy efficiency. Specifically tailored for machine learning, deep learning, computer vision, and associated domains, the TX2 boasts multiple connectivity interfaces and comprehensive software support. Consequently, it has found extensive use across diverse domains including unmanned aerial vehicles, robotics, intelligent cameras, and similar fields.The specific specifications of the NVIDIA TX2 used are detailed in Table 7.

**Table 7:** Detailed specifications of the Jetson TX2 embedded system.

Items	Specification
CPU	Dual-core NVIDIA Denver 2 64-Bit CPU Quad-Core ARM Cortex-A57 MPCore
GPU	256-core NVIDIA Pascal architecture GPU
Power	7.5 W/15 W
Memory	8GB 128-bit LPDDR4 Memory 1866 MHx
Storage	32 GB eMMC 5.1
Operating system	Linux for Tegra 28.1

We established the experimental setup on the NVIDIA TX2 platform using JetPack 4.5.1 and CUDA 10.1, and implemented PyTorch 1.8.1. Subsequently, the trained model was successfully deployed on the TX2 for real-time unmanned aerial vehicle (UAV) detection tests based on the UAVfly dataset scenarios. Detailed information regarding the average inference speed of various models processing individual frame images is presented in Table 8. The obtained outcomes highlight the model we proposed achieving a processing rate of 69 frames per second (FPS), demonstrating substantial potential to meet practical demands in UAV detection tasks.

**Table 8:** Embedded experiment results.

Model	mAP 0.5	Inference speeds (ms)	FPS
YOLOv5s	0.933	17.3	58
YOLOv4	0.927	34.4	29
YOLOv3	0.901	47.6	21
YOLOv5m	0.943	25.6	39
YOLOX-s	0.951	22.3	45
YOLOv7	0.958	28.6	35
YOLOv7-tiny	0.939	16.2	62
YOLOv5s-ngn	0.952	14.5	69

### Unmanned aerial vehicle detection results

**Figure 7:**
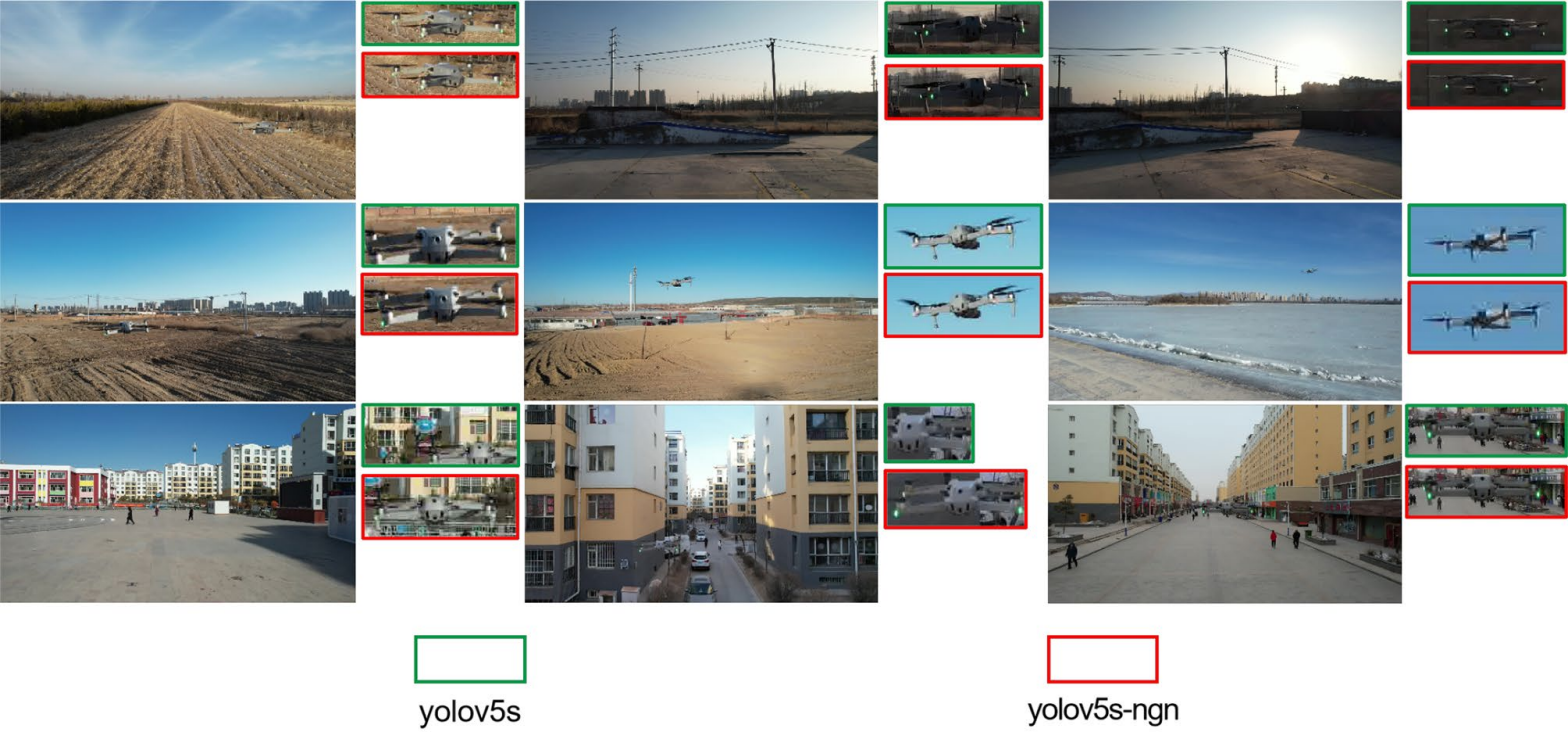
Detection performance.

Figure 7 demonstrates the detection performance of the original YOLOv5s model and the improved model on unmanned aerial vehicle (UAV) targets. Based on Fig. 7, it is noticeable that the enhanced model demonstrates heightened confidence in UAV detection outcomes, with anchor boxes displaying a strong alignment with UAV positions, thereby implying improved localization accuracy. The improved network model can accurately identify UAVs, therefore, the proposed lightweight network-based model can perform real-time detection on UAVs.

## Discussion

To address the challenge of achieving real-time detection among drones during operation, we propose a lightweight air-to-air drone detection model based on the YOLOv5s architecture. Initially, we achieved lightweight feature extraction by integrating the ${\text {CF2}}-{\text {MC}}+{\text {SPP}}$ lightweight feature extraction network into YOLOv5s. Simultaneously, we introduced an innovative feature fusion module (MG) and applied the EIoU loss function, aiming to enhance the detection accuracy while reducing the complexity of the YOLOv5s model. The optimized YOLOv5s model demonstrated outstanding performance on the UAVfly and Det-Fly datasets. Lastly, embedded experiments conducted on the NVIDIA TX2 platform revealed an average inference speed of only 14.5 ms per single-frame image. Despite YOLOv5s-ngn accomplishing real-time detection of air-to-air drones, it still faces limitations, such as the datasets’ inability to fully represent the actual operational environments of drones and challenges in distinguishing between multiple overlapping drone images. Our future work will involve expanding the dataset to encompass a more comprehensive range of real-world drone operating environments, including scenarios with multiple overlapping drones. Furthermore, our focus will extend to exploring model optimization techniques to achieve faster and more accurate drone detection, which will remain a significant focal point in our future endeavors.

## Data Availability

The datasets generated during and/or analysed during the current study are available at https://github.com/lucien22588/UAVfly.git.
